# Human *Toxoplasma gondii* infection in Nigeria: a systematic review and meta-analysis of data published between 1960 and 2019

**DOI:** 10.1186/s12889-020-09015-7

**Published:** 2020-06-06

**Authors:** Solomon Ngutor Karshima, Magdalene Nguvan Karshima

**Affiliations:** 1 Department of Veterinary Public Health and Preventive Medicine, University of Jos, PMB 2084, Jos, Nigeria; 2 Department of Parasitology and Entomology, Modibbo Adama University of Technology, Yola, Adamawa State Nigeria

**Keywords:** Geographical distribution, HIV patients, Normal individuals, Pregnant women, Prevalence, *Toxoplasma gondii*

## Abstract

**Background:**

Over 70% of the worlds’ population is infected by *Toxoplasma gondii*; a pathogen capable of causing cerebral toxoplasmosis in HIV patients and neonatal complications like miscarriage, chorioretinitis, hydrocephalus, cerebral calcification and foetal death in the third trimester of pregnancy. In spite of this, the burden of this zoonotic pathogen is poorly understood in Nigeria. The aim of the present study therefore, is to determine the burden of *T. gondii* among normal individuals, HIV patients and pregnant women as well as the distribution of the infection across Nigeria.

**Methods:**

Using the PRISMA guidelines, we conducted a systematic review and meta-analysis of data retrieved from six electronic databases (AJOL, Google Scholar, PubMed, Scopus, Science Direct and Web of Science). Pooled prevalence (PP) and heterogeneity were determined by the random-effects model and the Cochran’s Q-test respectively. The quality of each study and publication bias were assessed by the 9 point Joanna Briggs Institute Critical Appraisal Instrument and the Egger’s regression asymmetry test respectively, while the robustness of a pooled estimate was tested by the single study omission analysis.

**Results:**

Exactly 5834 of the 16,230 individuals examined for *T. gondii* infection by 50 studies across 17 Nigerian States were positive for the infection. Overall PP was 32.92% (95% CI: 27.89, 38.37), with a range of 14.41% (95% CI: 5.32, 33.54) to 86.82% (95% CI: 66.13, 95.69) across sub-groups. Pooled prevalence was significantly higher (*p* < 0.001) among pregnant women (40.25%; 95% CI: 33.19, 47.73) and HIV patients (31.68, 95% CI: 20.53, 45.41) than normal individuals (23.32, 95% CI: 17.25, 30.75). *T. gondii* prevalence declined by over 58% during the 59 years reviewed.

**Conclusion:**

*Toxoplasma gondii* infection is moderately prevalent in Nigeria. Highest prevalence estimates were observed among pregnant women and in the south-south region. For effective control of the disease in Nigeria, a holistic approach involving on-farm, environmental, public health and animal components are suggested.

## Background

*Toxoplasma gondii* is an obligate intracellular protozoan zoonotic pathogen of almost all warm-blooded animals including humans and birds [1]. The infection is worldwide in distribution and over 70% of the worlds’ population is infected [2, 3]. Domestic cats are the definitive hosts, and represent the main source of infection through oocysts passed in their faeces. The pathogen is currently a global problem which is present in every country of the world [4].

Human infection may result via several routes including contact with infected cats, the consumption of animal tissues infected by cysts of *T. gondii*, the ingestion of food or water contaminated with oocysts excreted in the faeces of cats, blood transfusion and intrauterine [5–7]. Transmission is influenced by factors such as environmental conditions, host immune status, cultural behaviour, individual’s hygienic practices, type of food and cooking methods [8, 9].

In immunocompetent individuals, *Toxoplasma* infection may be asymptomatic or self-limiting. However, immunocompromised conditions like HIV infection may alter the clinical course of *T. gondii* infection [10, 11]. HIV infection may cause reactivation of the asymptomatic *Toxoplasma* infection resulting in neurological signs like headache, disorientation, drowsiness, hemiparesis, reflex changes and convulsion [12–14]. About 25–50% of HIV immunocompromised individuals may show the signs of cerebral toxoplasmosis [15] and *Toxoplasma* infection is now a known cause of morbidity and mortality in people living with HIV and AIDS (PLWHA) [12].

*Toxoplasma* induced neonatal complications especially during third trimester of pregnancy may include miscarriage, chorioretinitis, hydrocephalus, cerebral calcification and foetal death [16, 17]. The risk of transmission of *Toxoplasma* infection from mother to child intrauterine is increased during the third trimester of pregnancy [18, 19]. Continental prevalence of *T. gondii* across the world ranged between 4.3–75.0% in Africa [20–23], 14.0–96.3% in Asia [24–26], 6.8–51.8% in Europe [27–29], 10.6–13.0% in North America [30–32] and 26.3–80.0% in South America [33–35].

Despite the association of toxoplasmosis with immunocompromised conditions and the increasing number of PLWHA and pregnant women in Nigeria, the nationwide prevalence and burden of *T. gondii* infection are poorly understood. In this study, we reported the burden of *T. gondii* infections among normal individuals, HIV patients and pregnant women in Nigeria. It is envisaged that the present finding will enable stakeholders and policy makers in the health sector to re-strategize on the control of toxoplasmosis, thus reducing the burden of the disease especially in Nigerian pregnant women.

## Methods

### Study protocol and literature search procedure

We conducted a systematic review and meta-analysis using the guidelines provided by Moher [36] for Preferred Reporting Items for Systematic Reviews and Meta-Analyses (PRISMA). Inclusion of data for quantitative synthesis was based on the PRISMA checklist (Additional file 1), and the infection of humans with *Toxoplasma gondii* was the outcome of interest. The review protocol was registered on PROSPERO International prospective register of systematic reviews with registration number CRD42019135416 and available from: http://www.crd.york.ac.uk/PROSPERO/display_record.php?ID=CRD42019135416.

Six electronic databases: African Journals OnLine (AJOL), Google Scholar, PubMed, Scopus, Science Direct and Web of Science were systematically searched between 1st June and 30th April 2020 for literature published on *T. gondii* infection in humans in Nigeria between 1960 and 2019. Additional studies were obtained through searching of list of references of retrieved studies and by contacting editors of Nigerian biomedical journals. Full articles with only visible online abstracts were requested from authors and editors of the publishing journals through phone calls or e-mails. The *MeSH* search string employed in PubMed was “toxoplasmosis” OR “*Toxoplasma*” OR “*Toxoplasma gondii*” OR “*Toxoplasma* infections” AND “Prevalence” OR “Seroprevalence” OR “Seroepidemiology” AND “Humans” OR “Healthy individuals” OR “HIV patients” OR “Pregnant women” AND “North-central” OR “North-eastern” OR “North-western” Or “South-eastern” OR “South-south” OR “South-western” AND “Nigeria”. Search on AJOL was carried out on journal by journal bases within biomedical journals indexed in the database. Due to the large volume of data in Google Scholar, the study customised article search in this database based on year of publication for ease of sorting articles.

### Requirements for inclusion

Studies were subjected to two stages of screening for either inclusion or exclusion. In the first stage, studies were screened by scanning through titles for exclusion of duplicates. Second stage screening involved detailed review of abstract and full text for removal of irrelevant studies and identification of relevant information. A study was considered for inclusion if it had the following characteristics: (i) it was carried out in Nigeria, (ii) it was published in English, (iii) it was carried out and published between January 1960 and December 2019, (iv) it was a cross sectional or prevalence study, (v) it stated the study location, (vi) it clearly stated the number of sample size and positive cases, (vii) it reported *T. gondii* in humans, and (viii) it stated the target population. Studies that did not meet these inclusion criteria and all unpublished articles were excluded.

### Quality assessment

The quality of each article analysed was assessed independently using the 9 point Joanna Briggs Institute (JBI) critical appraisal instrument for studies reporting prevalence data [37]. The JBI checklist posed nine questions viz.: (1) Was the sample frame appropriate to address the target population? (2) Were study participants recruited in an appropriate way? (3) Was the sample size adequate? (4) Were the study subjects and setting described in detail? (5) Was data analysis conducted with sufficient coverage of the identified sample? (6) Were valid methods used for the identification of the condition? (7) Was the condition measured in a standard, reliable way for all participants? (8) Was there appropriate statistical analysis? (9) Was the response rate adequate, and if not, was the low response rate managed appropriately (Additional file 2)? Answers to the aforementioned questions for individual studies were respectively assigned scores of 0 or 1 for no or yes answers, while U or NA were used when a study does not clearly answer the question or when the question was not applicable to the study. For a study to be included in the quantitative synthesis, it was required to have a minimum quality assessment score of 6 (66.7%); that is answering yes to at least 6 of the 9 questions on the checklist.

### Extraction of data

To ensure data validation and increase the likelihood of detecting errors, literature search, screening of articles, selection of articles for eligibility and data extraction were performed by both authors (SNK and MNK) independently. However, in cases of discrepancies, both authors crosschecked data simultaneously and discussed issues until consensus was reached. Data pulled out from each published study were name of author, the year the study was carried out, the year it was published, sample size, number of positive cases, study location, study design, method of diagnosis, diagnostic target and characteristics of study population. Where specified, the gender and ages of study population were also extracted and individuals within the age brackets ≤17 years of age were categorised as children while those ≥18 years of age were categorised as adults. Where the study year for any article was not stated, the year preceding its publication year was considered as the year it was conducted.

In our PICOS, we answered questions such as: (1) What is the burden of *Toxoplasma gondii* infection in normal individuals, HIV patients and pregnant women from Nigeria? (2) Is the burden greater among immunocompromised than normal individuals? (3) What is the distribution pattern of the infection across Nigeria? For the purpose of the present study, an individual was said to be infected with *T. gondii* only if the individual tested positive for the parasite by microscopic, serological or molecular techniques, normal individuals refers to individuals without any history of pregnancy or any immunocompromised conditions like HIV/AIDS and neoplasia, pregnancy refers to a state where a woman carried an embryo or foetus for a period of ±9 months and HIV patients refer to people living with HIV/AIDS. More so, for the purpose of our analysis, diagnostic methods like polymerase chain reaction (PCR) and immunochromatography which were utilized by only one study, latex agglutination test (LAT) which was utilized by only two studies and studies with unidentified methods of diagnosis were grouped as others.

### Data collation and analyses

Preliminary analyses including summations, subtractions, divisions, multiplications and estimation of percentages were conducted using Microsoft Excel. Statistical and meta-analyses were carried out using Graph-Pad Prism version 4.0 and Comprehensive Meta-Analysis version 3.0 respectively. Prevalence of individual studies was determined by expressing the proportion of positive cases of *T. gondii* infection and sample size as percentages.

### Pooling, sub-group and heterogeneity analyses

Pooled prevalence and their 95% Confidence Interval (CI) were estimated by the random-effects model [38]. Sub-group analyses were performed based on ages (Adult, children), gender (female, male), characteristics of study population (normal individuals, HIV patients and pregnant women) and geographic regions (North-central, north-east, north-west, south-east, south-south and south-west). Others were diagnostic methods (Dye test, ELISA, HAT, LAT, PCR, and RSAT), study period (1960–1975, 1976–1990, 1991–2005 and 2006–2019) as well as sample size (≤ 150, 151–300, 301–450 and > 450).

Heterogeneity among studies was evaluated using the Cochran’s Q-test while the percentage variation among studies due to heterogeneity was quantified using the formula *I*^2^ = 100 × (Q-*df*)/Q; where Q is Cochran’s heterogeneity statistic and *df* is the degree of freedom which is determined by subtracting one from the number of studies analysed. I-square values of 0, 25, 50 and 75% were considered no, low, moderate and substantive heterogeneities respectively [39, 40].

### Publication bias, sensitivity and meta-regression analyses

Publication bias (across-study bias) was examined by funnel plots while the statistical significance was assessed by the Egger’s regression asymmetry test [41]. The unbiased estimates were calculated using the Duval and Tweedie non-parametric ‘fill and trim’ linear random method [42]. The robustness of a pooled estimate was tested by the single study omission analysis, and a study was considered to have no influence on the pooled prevalence if the pooled estimate without it (i.e number of studies = 49) was within the 95% confidence limits of the overall pooled prevalence when number of studies equals 50 [43]. Meta-regression analysis was performed for different sub-groups including year of conduct of study, diagnostic methods, geographic regions as well as age, gender and characteristics of the study population to determine the possible sources of heterogeneity.

## Results

### Literature search and eligible studies

**Fig. 1:**
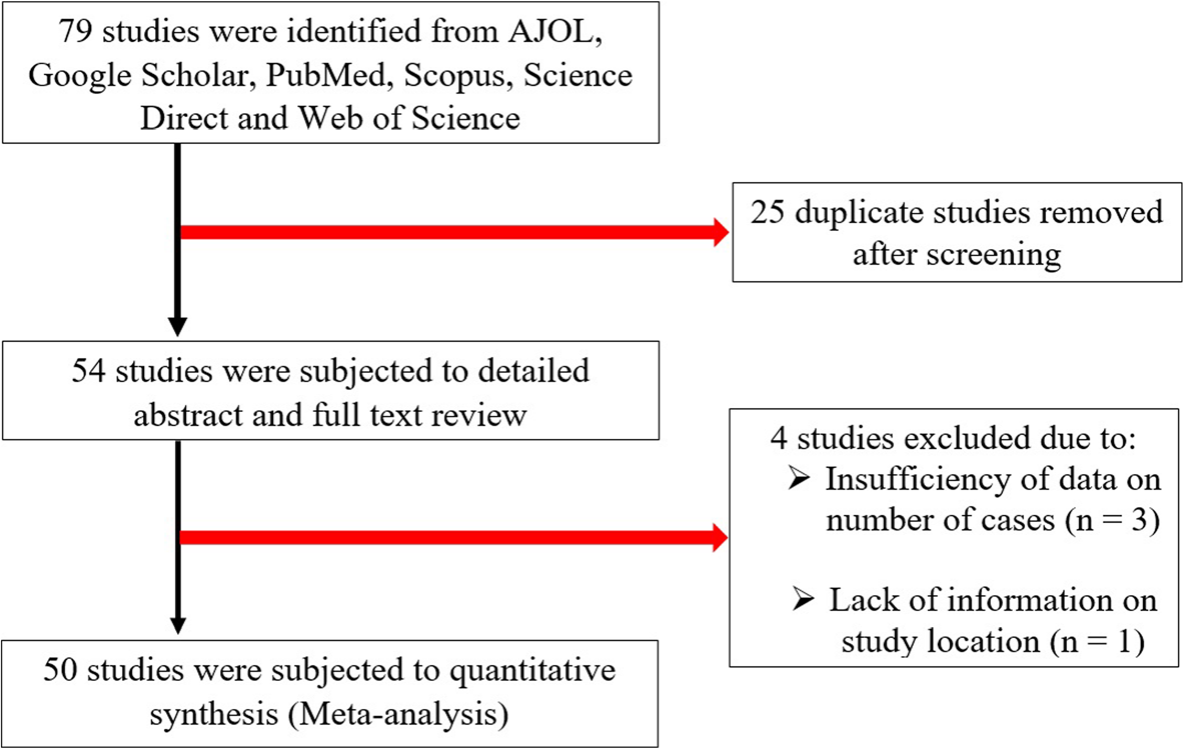
Flow diagram for the selection of eligible studies

The procedure for selection of eligible studies is presented in Fig. 1. A total of 79 studies resulted from the search of six electronic databases. Twenty five of the studies were duplicates and were removed after screening of titles. Fifty four of the studies were subjected to detailed review of abstract and full text. Four studies were thereafter removed for insufficiency of data on sample sizes and number of cases (*n* = 3) and lack of information on study location (*n* = 1). Fifty studies were subjected to the quantitative synthesis. None of the studies assessed for quality by the JBI critical appraisal instrument was excluded for lack of merit. Quality scores ranged between 6 and 8 (66.67–88.89%) of a total of 9 scores (Table 1 and Additional file 3).

**Table 1:** List and characteristics of eligible studies

Study year	Study location	Region	Method of Diagnosis	Diagnostic target	Characteristics of study population	Sample size	Cases	Prev. (%)	95% CI	QAS	Study Ref
2015	Osun	South-west	ELISA	IgG and IgM	Pregnant women	391	186	47.57	42.53–52.65	6	[44]
1999–04	Kwara	North-central	ELISA	Not stated	HIV-positive individuals	60	25	41.67	29.07–55.12	7	[45]
1989	Kaduna	North-west	HAT	Not stated	Pregnant women	834	329	39.45	36.11–42.86	8	[46]
2009	Lagos	South-west	–	IgG	HIV-positive individuals	460	230	50.00	45.34–54.66	7	[47]
2009	Lagos	South-west	ELISA	IgG	Pregnant women	179	73	40.78	33.51–48.36	7	[48]
2011	Sokoto	North-west	RSAT	IgG	HIV-positive individuals	84	22	26.19	17.20–36.93	6	[49]
2011	Sokoto	North-west	LAT	IgG	Abattoir workers	75	38	50.67	38.86–62.42	6	[50]
2012	Sokoto	North-west	RSAT	IgG	Pregnant women	173	48	27.75	21.22–35.05	6	[51]
2018	Oyo	South-west	ELISA	IgG and IgM	Blood donors	248	106	42.74	36.50–49.16	7	[52]
2011	Benue	North-central	ELISA	IgG	HIV-positive individuals	360	39	10.83	7.82–14.51	7	[53]
1985	Delta	South-south	Dye Test	Not stated	Pregnant women and blood donors	1650	972	58.91	56.49–61.30	7	[54]
2013	Oyo	South-west	PCR	DNA	Pregnant women	179	49	27.37	20.99–34.53	6	[55]
2008	Kaduna	North-west	ELISA	IgG and IgM	Pregnant women	374	112	29.95	25.35–34.87	8	[56]
2010	Lagos	South-west	ELISA	IgG and IgM	Pregnant women	276	111	40.22	34.38–46.26	6	[57]
2016/17	Akwa Ibom	South-south	ELISA	IgG	Abattoir workers	339	189	55.75	50.29–61.12	7	[58]
1993	Oyo	South-west	–	IgG	Lymphoid neoplasia patients	162	44	27.16	20.48–34.70	6	[59]
2018	Ogun	South-west	RSAT	IgG and IgM	Young adults with history of ocular infections	150	3	2.00	0.41–5.73	8	[60]
2011	Borno	North-east	ELISA	IgG	HIV positive individuals	190	42	22.11	16.42–28.68	7	[61]
2013/14	Lagos	South-west	LAT	Not stated	Primary school children	382	91	23.83	19.64–28.42	7	[62]
2016	Kano	North-west	ELISA	IgM	Obstetric patients	320	24	7.50	4.86–10.95	6	[63]
1960	Kano	North-west	Dye Test	Not stated	Chorioretinitis patients	17	15	88.24	63.56–98.54	7	[64]
2011/12	Edo	South-south	ELISA	IgG and IgM	Psychotic individuals	280	90	32.14	26.71–37.96	7	[65]
2008	Borno	North-east	ELISA	IgG	Normal individuals	180	43	23.89	17.9–30.8	7	[66]
2011	Abuja	North-central	ELISA	IgG and IgM	HIV-positive individuals	341	30	8.80	6.0–12.3	6	[67]
2014	Borno	North-east	ELISA	IgG and IgM	Pregnant women	360	176	48.89	43.6–54.2	8	[68]
2013	Rivers	South-south	ELISA	IgG and IgM	Pregnant women	288	189	65.63	59.8–71.1	8	[69]
2018	Edo	South-south	ICT	IgG and IgM	HIV-positive and healthy individuals	1500	113	7.53	6.3–9.0	6	[70]
2008	Kaduna	North-west	ELISA	IgG and IgM	HIV-positive and healthy individuals	219	78	35.62	29.3–42.4	8	[71]
1980	Kano	North-west	HAT	Not stated	Pregnant women and male patients	20	7	35.00	15.4–59.2	7	[72]
Study year	Study location	Region	Method of Diagnosis	Diagnostic targets	Characteristics of study population	Sample size	Cases	Prev. (%)	95% CI	QAS	Study Ref
2009–12	Lagos	South-west	ELISA	IgG	HIV-positive individuals	242	100	41.32	35.05–47.81	8	[73]
2014	Lagos	South-west	ELISA	IgG and IgM	HIV positive and normal individuals	840	286	34.05	30.84–37.36	7	[74]
1995	Benue	North-central	–	IgG	Pregnant women	606	265	43.73	39.74–47.78	8	[75]
1991	Oyo	South-west	Dye Test	IgG	Pregnant women	352	273	77.66	72.83–81.81	8	[76]
2008	Lagos	South-west	–	Not stated	HIV-positive individuals	400	14	3.50	1.93–5.80	6	[77]
2016	Rivers	South-south	ELISA	IgG	Healthy individuals	800	329	41.13	37.69–44.62	7	[78]
2009	Lagos	South-west	–	IgG	HIV-positive individuals	83	71	85.54	76.11–92.30	7	[79]
1984	Plateau	North-central	HAT	Not stated	Pregnant women and other hospital patients	210	48	22.86	17.36–29.14	6	[80]
2010	Lagos	South-west	ELISA	IgG	HIV-positive individuals	380	206	54.21	49.05–59.30	7	[81]
2013	Borno	North-east	ELISA	IgG	Pregnant women	90	20	22.22	14.13–32.21	6	[82]
2015	Lagos	South-west	HAT	IgG and IgM	HIV-positive and normal individuals	65	2	3.08	0.37–10.68	6	[83]
1986	Niger	North-central	–	Not stated	Hospital patients	176	139	78.98	72.21–84.75	6	[84]
2016	Osun	South-west	ELISA	IgG and IgM	Pre-school aged children	272	19	6.99	4.26–10.69	7	[85]
1980	Oyo	South-west	ELISA	Not stated	Children	66	11	16.67	8.63–27.87	6	[86]
2004	Plateau	North-central	–	IgG	HIV-positive individuals	363	115	31.68	26.92–36.74	7	[87]
2006	Plateau	North-central	ELISA	IgG	Normal individuals	144	30	20.83	14.52–28.39	6	[88]
2011	Abuja	North-central	ELISA	IgG	Pregnant, HIV-positive and normal individuals	216	68	31.48	25.35–38.13	8	[89]
1969	Oyo	South-west	Dye Test	Not stated	Normal individuals	6	5	83.33	35.88–99.58	6	[90]
2016	Rivers	South-south	ELISA	IgG and IgM	Pregnant women	213	58	27.23	21.37–33.73	7	[91]
2014	Kano	North-west	ELISA	IgG and IgM	HIV-positive pregnant women	273	93	34.07	28.46–40.02	8	[92]
2015	Edo	South-south	ELISA	IgG and IgM	HIV-positive individuals	342	208	60.82	55.42–66.03	8	[93]

*CI* Confidence interval; *ELISA* Enzyme linked immunosorbent assay; *HAT* Haemagglutination test, *ICT* Immunochromatographic test; *IgG* Immunoblobulin G; *IgM* Immunoglobulin M; *LAT* Latex agglutination test; *PCR* Polymerase chain reaction; *QAS* Quality assessment score; *RSAT* Rapid slide agglutination test; *Prev.* Prevalence

### Characteristics of eligible studies

Table 1 shows the characteristics of the eligible studies. Fifty studies examined 16,230 individuals for *Toxoplasma gondii* infection among Nigerians and reported prevalence rates ranging between 2.00 and 88.24%. Nine, 4, 10, 8 and 19 of the studies were reported in the north-central, north-eastern, north-western, south-south and the south-western regions respectively. Three studies utilized rapid slide agglutination test (RSAT) for diagnosis, 4 each were diagnosed using dye and haemagglutination tests, 28 of the studies utilized enzyme linked immunosorbent assay (ELISA) while 11 studies utilized other methods (immunochromatographic test 1, LAT 2, PCR 1 and unidentified tests 7). One study each targeted *Toxoplasma***-**immunoglobulin M (IgM) and deoxyribonucleic acid (DNA), 20 studies targeted *Toxoplasma*-immunoglobulin G (IgG), 17 targeted both IgG and IgM, while 11 studies failed to state their diagnostic targets. Two studies were reported between 1960 and 1975, 6 between 1976 and 1990, 5 between 1991 and 2005 and 37 between 2006 and 2019. Twelve, 18, 13 and 7 of the studies had sample sizes of ≤150, 151–300, 301–450 and > 450 respectively.

### Spatial distribution of eligible studies

**Fig. 2:**
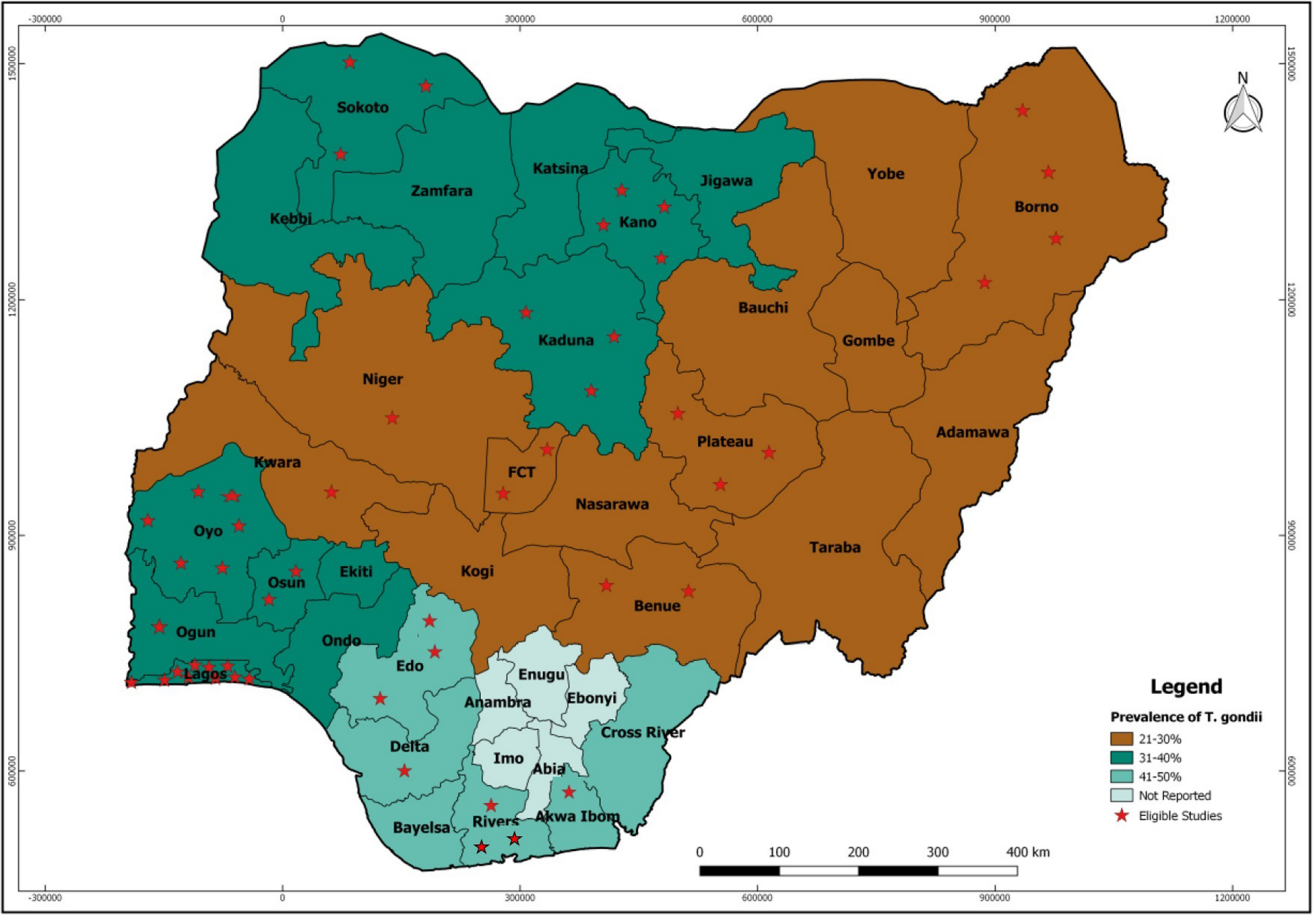
Regional prevalence of *T. gondii* and distribution of eligible studies in Nigeria

The 50 studies were reported across 5 of the 6 regions of Nigeria as presented in Fig. 2. The nine (18.00%) studies reported across the north-central region were distributed as follow: one each in Kwara and Niger States, 2 each in Abuja and Benue State, and 3 in Plateau State. Four (8.00%) studies were reported in Borno State, North-east Nigeria while the 10 (20.00%) studies reported across the north-west region were from Kaduna and Sokoto (3 studies each) and 4 from Kano. One study each was reported from Akwa Ibom and Delta States and 3 each from Edo and Rivers States totally 8 (16.00%) studies from the south-south while the 19 (38.00%) studies distributed across the south-west; 1, 2, 6 and 10 were from Ogun, Osun, Oyo and Lagos States respectively. No study was reported in the south-east region.

### Pooled prevalence and heterogeneity analysis

**Fig. 3:**
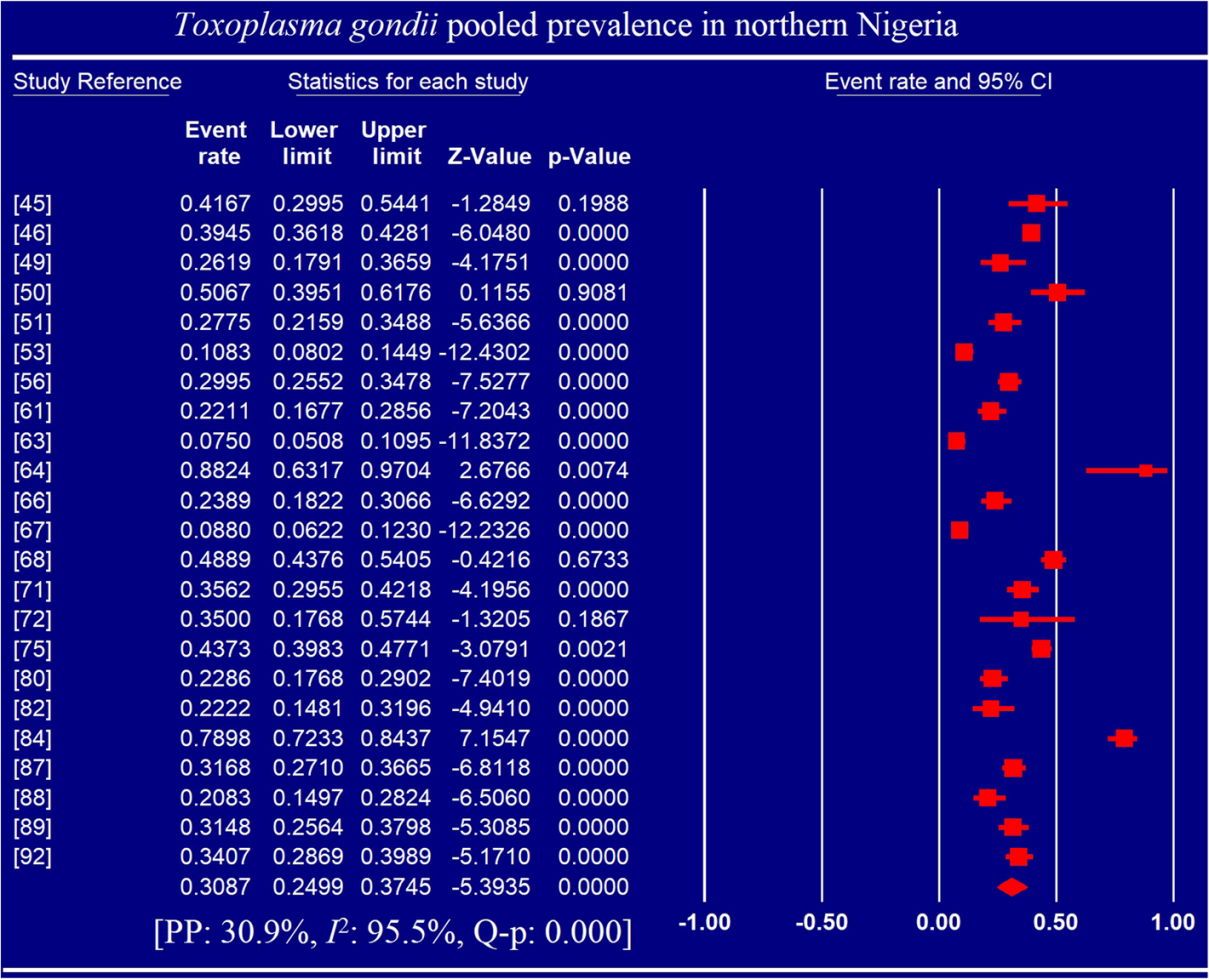
Forest plot for the prevalence of *Toxoplasma gondii* infection in northern Nigeria

**Fig. 4:**
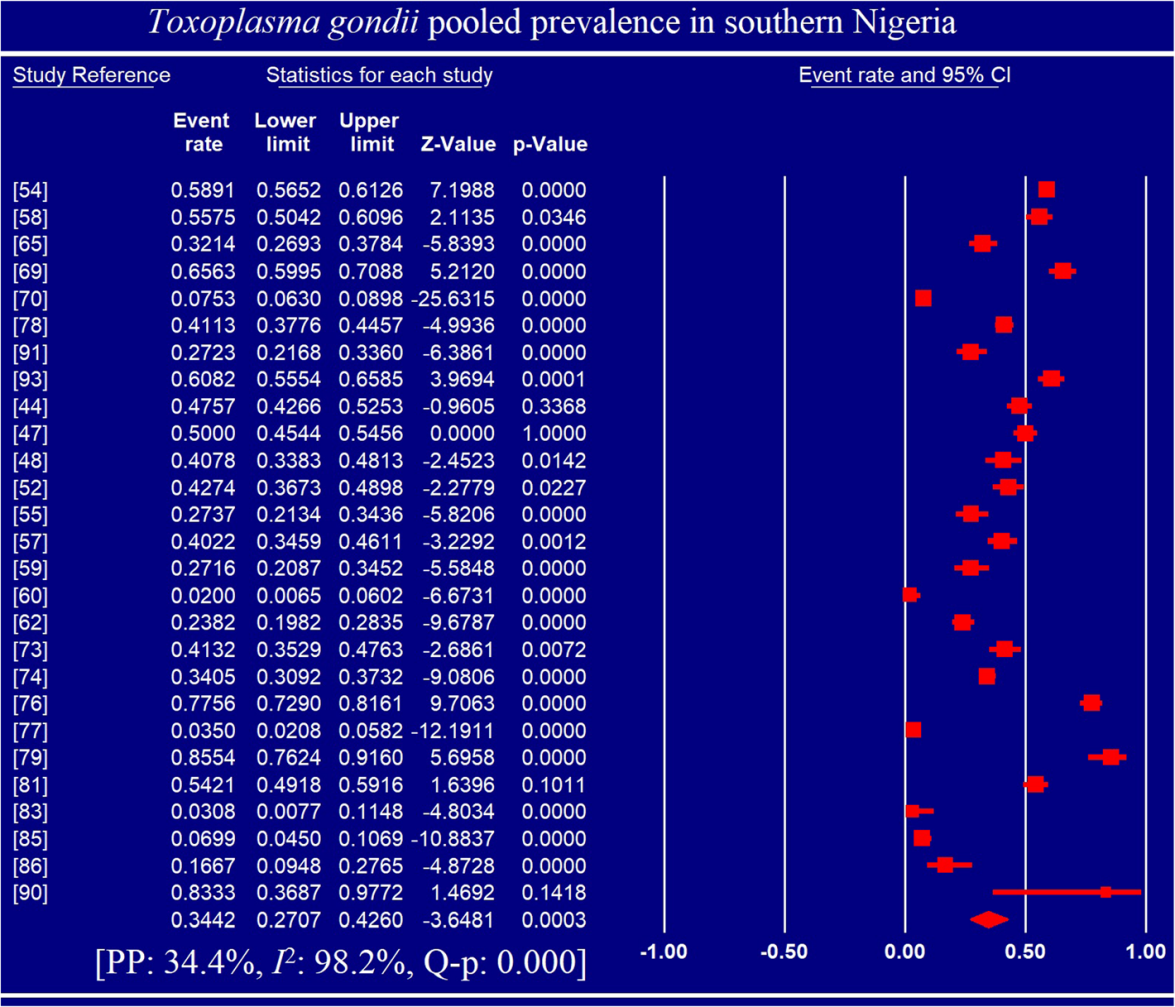
Forest plot for the prevalence of *Toxoplasma gondii* infection in southern Nigeria

Pooled prevalence and heterogeneities are presented in Tables 2 and 3 as well as Figs. 3 and 4. Fifty studies reported 5834 positive cases of *Toxoplasma gondii* infection among 16,230 individuals examined from 17 Nigerian States. Overall PP was 32.92% (95% CI: 27.89, 38.37; Q-*p* < 0.001) with ranges of 28.58 (95% CI: 16.28, 45.17; Q-*p* < 0.001) to 41.00% (95% CI: 25.61, 58.37; Q-*p* < 0.001) across geographic regions, 14.41% (95% CI: 5.32, 33.54; Q-p < 0.001) to 74.29% (95% CI: 57.49, 86.06; Q-*p* < 0.001) across methods of diagnosis, 28.75 (95% CI: 23.44, 34.71; Q-*p* < 0.001) to 86.82% (95% CI: 66.13, 95.69; Q-p: 0.760) across study periods and 30.22 (95% CI: 19.87–43.07; Q-*p* < 0.001) to 36.64% (95% CI: 24.15, 51.23; Q-p < 0.001) across sample size.

**Table 2:** Pooled prevalence of *T. gondii* infection in humans in Nigeria based of sub-groups

Variables	No. of Studies	Pooled Estimates			(95% CI)	Heterogeneity			Meta-regression	
		Sample size	Cases	Prev. (%)		Q-value	***I***^**2**^ (%)	Q-***p***	***P***- value	OR (95% CI)
Region										
South-west	19	5133	1880	31.70	23.86, 40.74	618.15	97.09	< 0.001	0.756	−0.10 (−0.80, 0.61)
South-south	8	5412	2148	41.00	25.61, 58.37	825.41	99.15	< 0.001		−0.15 (−1.10, 0.81)
North-west	10	2389	766	32.52	25.03, 41.03	116.68	92.29	< 0.001		0.10 (−0.59, 0.80)
North-east	4	820	281	28.58	16.28, 45.17	58.84	94.90	< 0.001		0.41 (−0.32, 1.14)
North-central	9	2476	759	29.58	18.54, 43.66	313.91	97.45	< 0.001		Reference
MOD										
RSAT	3	407	73	14.41	5.32, 33.54	23.60	91.53	< 0.001	< 0.001	−2.87 (−4.23, −1.51)
Others	11	4386	1169	34.79	21.32, 51.24	731.36	98.63	< 0.001		−1.76 (− 2.81, −0.71)
HAT	4	1129	386	23.71	12.85, 39.58	35.54	91.56	< 0.001		−2.34 (− 3.62, −1.07)
ELISA	28	8283	2941	31.62	26.39, 37.37	740.87	96.36	< 0.001		−1.90 (−2.88, −0.92)
Dye Test	4	2025	1265	74.29	57.49, 86.06	46.33	93.53	< 0.001		Reference
Study period										
2006–2019	37	11,708	3586	28.75	23.44, 34.71	1420.79	97.47	< 0.001	0.003	−2.76 (−4.48, −1.05)
1991–2005	5	1543	722	44.60	27.46, 63.13	172.73	97.68	< 0.001		−2.07 (−3.92, −0.23)
1976–1990	6	2956	1506	41.93	27.81, 57.51	218.12	97.71	< 0.001		−2.19 (−4.01, −0.36)
1960–1975	2	23	20	86.82	66.13, 95.69	0.09	0.00	0.760		Reference
Sample size										
> 450	7	6690	2524	36.64	24.15, 51.23	721.84	99.17	< 0.001	0.913	0.19 (−0.65, 1.03)
301–450	13	4704	1663	30.22	19.87, 43.07	790.96	98.48	< 0.001		−0.09 (− 0.81, 0.63)
151–300	18	3976	1398	33.68	27.00, 41.08	354.78	95.21	< 0.001		0.06 (−0.62, 0.73)
≤ 150	12	860	249	32.59	18.93, 50.04	160.68	93.15	< 0.001		Reference
Overall	**50**	**16,230**	**5834**	**32.92**	**27.89, 38.37**	**2071.70**	**97.64**	< **0.001**		

*CI* Confidence interval; *ELISA* Enzyme linked immunosorbent assay; *HAT* Haemagglutination test, *LAT* Latex agglutination test; *PCR* Polymerase chain reaction; *RSAT* Rapid slide agglutination test; *I*^2^ Inverse variance index; *MOD* Method of diagnosis; *OR* Odds Ratio; Prev. Prevalence; *Q-p* Cochran’s p-value

**Table 3:** Pooled prevalence of *Toxoplasma gondii* infections in relation to age, gender and characteristics of study population

Variables	No. of Studies	Pooled Estimates			(95% CI)	Heterogeneity			Meta-regression	
		Sample size	Cases	Prev. (%)		Q-value	***I***^**2**^ (%)	Q-***p***	***P***- value	OR (95% CI)
Age*										
Children	7	1365	551	18.52	6.02, 44.63	342.16	98.25	< 0.001	0.451	0.37 (−0.59, 1.33)
Adults	7	2790	1224	36.93	29.04, 45.59	106.08	94.34	< 0.001		Reference
Total	**14**	**4155**	**1775**	**29.55**	**21.19, 39.55**	**448.80**	**97.10**	**< 0.001**		
Gender								< 0.001		
Male	15	2391	1052	31.85	21.79, 43.94	353.07	96.04	< 0.001	0.835	−0.07 (−0.70, 0.57)
Female	22	4263	1667	30.61	22.95, 39.51	620.71	96.62	< 0.001		Reference
Total	**37**	**6654**	**2719**	**31.23**	**25.15, 38.02**	**981.86**	**96.33**	< **0.001**		
Target Population										
Pregnant women	20	6560	2990	40.25	33.19, 47.73	600.05	96.83	< 0.001	0.034	0.35 (−0.21, 0.91)
Healthy individuals	11	1372	313	23.32	17.25, 30.75	70.24	85.76	< 0.001		−0.49 (−1.16, 0.18)
HIV patients	16	4061	1163	31.68	20.53, 45.41	767.23	98.05	< 0.001		Reference
Total	**47**	**11,993**	**4466**	**32.94**	**27.61, 38.74**	**1614.29**	**97.15**	< **0.001**		

^*^(*P* = 0.022); *CI* Confidence interval; *HIV* Human immunodeficiency virus; *I*^2^ Inverse variance index; *MOD* Method of diagnosis; *OR* Odds Ratio; *Prev.* Prevalence; *Q-p* Cochran’s p-value

Pooled prevalence of *T. gondii* infection among adults and children were 36.93% (95% CI: 29.04, 45.59; Q-p < 0.001) and 18.52% (95% CI: 6.02, 44.63; Q-p < 0.001) respectively. Gender-based PP were 30.61% (95% CI: 22.95, 39.51; Q-p < 0.001) and 31.85% (95% CI: 21.79, 43.94; Q-p < 0.001) for females and males respectively. Pooled prevalence reported in both HIV-patients 31.68% (95% CI: 20.53, 45.41; Q-p < 0.001) and pregnant women 40.25% (95% CI: 33.19, 47.73; Q-p < 0.001) were significantly higher (p < 0.001) than that among normal individuals 23.32% (95% CI: 17.25, 30.75; Q-p: 0.001). Overall heterogeneity was 97.64% with a range of 85.76 to 99.17% (Tables 2 and 3).

### Publication bias, sensitivity and meta-regression analyses

**Fig. 5:**
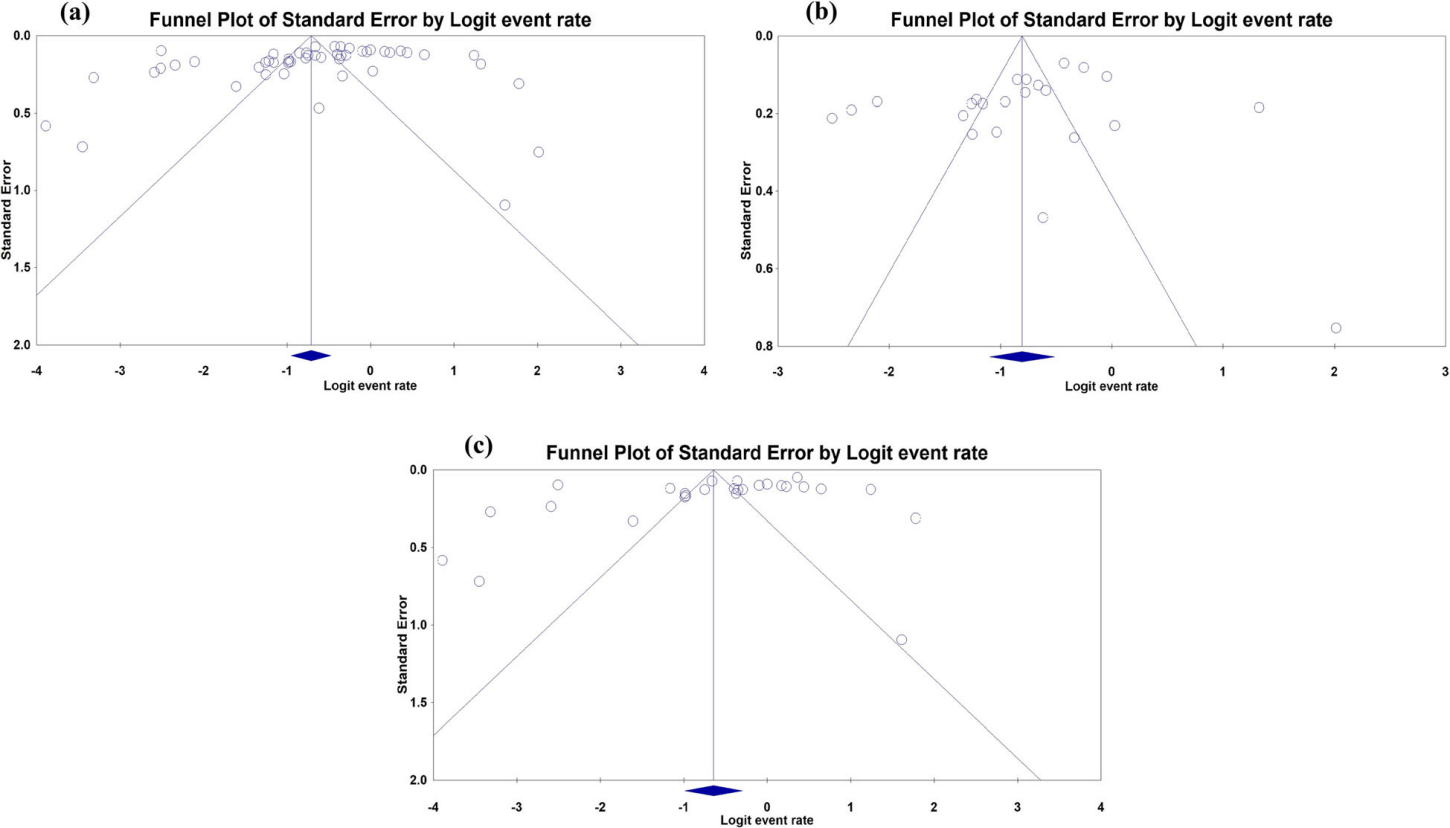
Funnel plots of standard error by logit event rate for pooled prevalence of *T. gondii* in **a** Nigeria, **b** northern Nigeria and **c** southern Nigeria

The funnel plots (Fig. 5) and their respective bias coefficients for studies published in Nigeria as a whole (b: -3.48; 95% CI: − 7.53, 0.58; *p*: 0.091), northern (b: -3.10; 95% CI: − 7.90, 1.71; *p*: 0.194) and southern regions (b: -5.03; 95% CI: − 11.05, 0.99; *p*: 0.098) suggest insignificant publication bias. No outlying study capable of causing publication bias was identified and removed by the Duval and Tweedie’s trim and fill method (Additional file 4). As in Additional file 5, the sensitivity tests showed that all single-study omission estimates were within the 95% CI of the overall PP.

Meta-regression analysis (Tables 2 and 3) suggests that geographic regions (Q: 1.89; *df*: 4; *p*: 0.756), sample size (Q: 0.53; *df*: 3; *p*: 0.913), age (Q: 0.57; *df*: 1; *p*: 0.451) and gender of participants (Q: 0.04; *df*: 1; *p*: 0.835) were unlikely to be the sources of heterogeneity in the present analysis. However, the year of conduct of the studies (Q: 13.72; *df*: 3; *p*: 0.003), methods of diagnosis employed by the studies (Q: 20.65; *df*: 4; *p* < 0.001) and characteristics of study population (Q: 6.00; *df*: 2; *p*: 0.034) might be the possible causes of the heterogeneity in our analysis.

## Discussion

Adequate understanding of the nationwide burden of toxoplasmosis is essential for effective prevention and control of the disease and its associated complications in immunocompromised individuals. Here, data from individual surveillance studies were harmonised across Nigeria with the sole aim of providing epidemiological information that may serve as a guide for disease monitoring and control in Nigeria.

We observed an overall PP of 32.92% which is consistent with a global report of 34.2% for middle income countries [94]. Our finding is however, higher than the 27.9% reported from Mexico [95] but lower than the 74.7% prevalence reported from Ethiopia [96]. *T. gondii* infection also showed geographical variations across Nigeria with the highest prevalence observed in the south-south region. Corroborating our finding, geographical variations within countries have been documented elsewhere. For instance, in sub-Saharan Africa, studies from Ethiopia reported prevalence of 68.4, 81.4 and 88.2% in the north-western, central and southern regions respectively [19, 22, 97]. More so, in Asia, studies from China documented prevalence of 8.4, 10.3, 12.3 and 21.6% in Anhui, Shanghai, Jilin and Yunnan provinces respectively [98–101]. Differences in weather conditions, eating habits, levels of environmental contamination with *T. gondii* oocysts, personal hygiene and human-cat contacts [96, 102] may be possible explanations for these variations across regions and countries.

The majority of the studies included in the analysis were diagnosed using ELISA. This may be due to the rapidity and accuracy of the method, convenience, ease of use, high sensitivity and specificity, cost effectiveness and the global acceptability of the test [103–105]. Studies diagnosed using the dye test recorded the highest disease prevalence probably due to its high specificity and sensitivity [106]. The serological tests conducted by the individual studies analysed targeted IgG (test for convalescent infection), IgM (test for recent or active infections) and their combination. Interestingly, 85% of the studies conducted on pregnant women detected IgM to identify activeness of infection which is a significant decision making stage to ameliorate or prevent congenital consequences based on stage of pregnancy.

The study showed over 58% decline in the PP of *T. gondii* in Nigeria during the 59 years under review (1960–2019). The decline in recent years could have resulted from controlled movement of cats, improved living standards, life styles and feeding habits that influence the transmission of this pathogen from cats and food animals to man. Other factors that may be responsible for the variations in *T. gondii* prevalence across study periods may include skills of the researchers involved as well as the sensitivity and specificity of the diagnostic methods employed by the individual studies. The majority of the studies were published within the twenty-first century in agreement with global report on *T. gondii* [94]. Increased interest on the pathogen arising from its association with severe complications in immunocompromised conditions like HIV infection, neoplasia and pregnancy may be a possible explanation.

The prevalence of *T. gondii* infection was higher in adults than children concurring reports from Mozambique [107] and Thailand [108]. This may be explained by the habits of eating undercooked roasted meat outside the home and possible intimacy of adults with pet cats. Corroborating a report from China [101], the present study observed a significantly higher prevalence in males than females. Contrary to our finding, Al-Qurashi et al. [7], Domingos et al. [107] and Tegegne et al. [109] reported higher prevalence in females in Saudi Arabia, Mozambique and Ethiopia, respectively.

Prevalence of *T. gondii* showed substantive heterogeneity among eligible studies reported across Nigeria. Meta-regression analysis suggests that this high heterogeneity might be from sources including years in which the studies were conducted, methods of diagnosis employed by the individual studies, characteristics of the study population and not from study locations, sample sizes, as well as age and gender of participants. The sensitivity tests showed that all single-study omission estimates were within the 95% CI of the overall prevalence, suggesting that the pooled prevalence of *T. gondii* was not substantially influenced by any single study. No significant publication bias was observed at alpha level of 0.05 with either Egger’s test or Duval and Tweedie’s method. The findings of the publication bias, sensitivity test and meta-regression substantiate the validity and reliability of the present analysis.

The PP of 40.25% observed among Nigerian pregnant women is higher than the range of 6.1–25.0% reported in countries like China [102, 110], Thailand [108] and Mexico [111]. The high prevalence among pregnant women is of major concern particularly in the ability of *T. gondii* to undergo intrauterine transmission and induce neonatal complications such as miscarriage, chorioretinitis, hydrocephalus, cerebral calcification and foetal death during pregnancy [16, 17]. *T. gondii* PP in HIV-patients in Nigeria was 31.68% concurring with reports of 36.3 and 38.0% from Thailand [112] and South Africa [113] respectively. The present finding is however; grossly lower than reports of 87.45% [17] and 90.0% [114] from Ethiopia. Another major concern with this finding is the risk of HIV changing the asymptomatic course of *T. gondii* in these individuals to a severe cerebral toxoplasmosis which is manifested by headache, disorientation, drowsiness, hemiparesis, reflex changes and convulsion [12–14].

The public health implications of the present finding in a developing country like Nigeria with a collapse primary health care system which is supposed to take care of the health of the rural majority are increased morbidity and associated complications in pregnant women and PLWHA. Two approaches are pertinent to the control of *T. gondii* in Nigeria. First, reviewing disease control programmes in Nigeria to include staging of pregnancy and IgG avidity test particularly in *Toxoplasma*-seropositive pregnant women for possible treatment to prevent congenital consequences and the restriction of cat movement. Second, stakeholders in the area of veterinary public health can also ensure on-farm good agricultural practices as well as standardized veterinary meat inspection to curtail the zoonotic transmission of this pathogen from companion and food animals to man.

The present study has several limitations despite its contribution to knowledge. We could not include some potentially relevant studies which would have added to the understanding of *T. gondii* infection in Nigeria due to insufficiency of data. A whole region (south-east) was not represented in the analysis because no study was published from the region. Studies analysed were published from only 17 of the 36 States in Nigeria including the Federal Capital Territory and were concentrated in the south-west (*n* = 19). Over 95% of the studies included in the analysis relied on serological methods of diagnosis which are unreliable in immunocompromised people [42]. This suggests a possible underestimation of the PP reported by the present study in immunocompromised people in Nigeria. The substantive heterogeneity observed among studies indicates variations across studies which could be due to several factors including year of conduct of study, methods of diagnosis and characteristics of study population. This suggests that the present finding may not represent an absolute *T. gondii* situation in Nigeria, but may provide a guide for disease control policies and the direction for future studies.

## Conclusion

*T. gondii* is moderately prevalent in Nigeria especially in the south-south region and among pregnant women and HIV-patients. The study showed over 58% decline in the PP of *T. gondii* during the 59 years reviewed (1960–2019). Age was a determining factor in the prevalence of *T. gondii* infection in Nigeria. To effectively control the disease, a holistic approach involving on-farm, environmental, public health and animal components are suggested.

## Supplementary information

**Additional file 1.** Preferred Reporting Items for Systematic Reviews and Meta-Analyses (PRISMA) checklist.

**Additional file 2.** JBI critical appraisal instrument for studies reporting prevalence data.

**Additional file 3.** Quality assessment scores for eligible studies.

**Additional file 4.** Results for Egger regression analysis.

**Additional file 5.** Results for single study omission analysis.

## Data Availability

The data supporting the conclusion of this article are all included in the article and Additional files 1-5.

## Abbreviations

AIDS: Acquired immunodeficiency virus
AJOL: African Journals OnLine
CI: Confidence interval
*df*: degree of freedom
ELISA: Enzyme linked immunosorbent assay
HAT: Haemagglutination test
HIV: Human immunodeficiency virus
ICT: Immunochromatographic test
*I*^2^: Inverse variance index
LAT: Latex agglutination test
MOD: Method of diagnosis
PCR: Polymerase chain reaction
PLWHA: People living with HIV/AIDS
PP: Pooled prevalence
Prev: Prevalence
PRISMA: Preferred Reporting System for Systematic Reviews and Meta-Analyses
Q: Cochran’s heterogeneity statistic
Q-p: Cochran’s *p*-value
RSAT: Rapid slide agglutination test
