# Supplementary material for: Human Toxoplasma gondii infection in Nigeria: a systematic review and meta-analysis of data published between 1960 and 2019
Source: BMC Public Health. 2020 Jun 6;20:877. doi: 10.1186/s12889-020-09015-7 (PMC7276081; doi:10.1186/s12889-020-09015-7)
Supplement: Supplementary file 3 — Additional file 3. Quality assessment scores for eligible studies. [file 12889_2020_9015_MOESM3_ESM.docx]

**Additional file 3:** Quality assessment scores for eligible studies

| **Study Name [Reference No.]** | **Q1** | **Q2** | **Q3** | **Q4** | **Q5** | **Q6** | **Q7** | **Q8** | **Q9** | **Overall score X/9**  **(% Score)** |
| --- | --- | --- | --- | --- | --- | --- | --- | --- | --- | --- |
| Adeyemi et al., 2018 [65] | 1 | 0 | 0 | 1 | 1 | 1 | 1 | 1 | NA | 6 (66.7) |
| Adepoju et al., 2007 [66] | 1 | 1 | 0 | 1 | 1 | 1 | 1 | 1 | NA | 7 (77.8) |
| Aganga et al., 1990 [67] | 1 | 1 | 1 | 1 | 1 | 1 | 1 | 1 | NA | 8 (88.9) |
| Akanmu et al., 2010 [68] | 1 | 1 | 0 | 1 | 1 | 1 | 1 | 1 | NA | 7 (77.8) |
| Akinbami et al., 2010 [69] | 1 | 1 | 0 | 1 | 1 | 1 | 1 | 1 | NA | 7 (77.8) |
| Alayande et al., 2012a [70] | 1 | 0 | 0 | 1 | 1 | 1 | 1 | 1 | NA | 6 (66.7) |
| Alayande et al., 2012b [71] | 1 | 0 | 0 | 1 | 1 | 1 | 1 | 1 | NA | 6 (66.7) |
| Alayande et al., 2013 [72] | 1 | 0 | 0 | 1 | 1 | 1 | 1 | 1 | NA | 6 (66.7) |
| Amoo et al., 2019 [73] | 1 | 1 | 0 | 1 | 1 | 1 | 1 | 1 | NA | 7 (77.8) |
| Amuta et al., 2012 [74] | 1 | 1 | 0 | 1 | 1 | 1 | 1 | 1 | NA | 7 (77.8) |
| Arene, 1986 [75] | 0 | 1 | 1 | 1 | 1 | 1 | 1 | 1 | NA | 7 (77.8) |
| Awobode and Olubi, 2014 [76] | 1 | 0 | 0 | 1 | 1 | 1 | 1 | 1 | NA | 6 (66.6) |
| Bata et al., 2009 [77] | 1 | 1 | 1 | 1 | 1 | 1 | 1 | 1 | NA | 8 (88.9) |
| Deji-Agboola et al., 2011 [78] | 1 | 0 | 0 | 1 | 1 | 1 | 1 | 1 | NA | 6 (66.7) |
| Ekanem et al., 2018 [79] | 1 | 0 | 1 | 1 | 1 | 1 | 1 | 1 | NA | 7 (77.8) |
| Ekweozor et al., 1994 [80] | 1 | 1 | 0 | 1 | 0 | 1 | 1 | 1 | NA | 6 (66.7) |
| Enitan et al., 2019 [81] | 1 | 1 | 1 | 1 | 1 | 1 | 1 | 1 | NA | 8 (88.9) |
| Goni et al., 2012 [82] | 1 | 1 | 0 | 1 | 1 | 1 | 1 | 1 | NA | 7 (77.8) |
| Gyang et al., 2015 [83] | 1 | 1 | 0 | 1 | 1 | 1 | 1 | 1 | NA | 7 (77.8) |
| Ibrahim et al., 2017 [84] | 1 | 0 | 0 | 1 | 1 | 1 | 1 | 1 | NA | 6 (66.7) |
| Iverson et al., 1960 [85] | 1 | 1 | 0 | 1 | 1 | 1 | 1 | 1 | NA | 7 (77.8) |
| James et al., 2013 [86] | 1 | 1 | 0 | 1 | 1 | 1 | 1 | 1 | NA | 7 (77.8) |
| Kamani et al., 2009 [87] | 1 | 1 | 0 | 1 | 1 | 1 | 1 | 1 | NA | 7 (77.8) |
| Mirabeau and Ebikade, 2012 [88] | 1 | 0 | 0 | 1 | 1 | 1 | 1 | 1 | NA | 6 (66.7) |
| Nasir et al., 2015 [89] | 1 | 1 | 1 | 1 | 1 | 1 | 1 | 1 | NA | 8 (88.9) |
| Oboro et al., 2016 [90] | 1 | 1 | 1 | 1 | 1 | 1 | 1 | 1 | NA | 8 (88.9) |
| Ogefere et al., 2019 [91] | 1 | 0 | 0 | 1 | 1 | 1 | 1 | 1 | NA | 6 (66.7) |
| Ogoina et al., 2013 [92] | 1 | 1 | 1 | 1 | 1 | 1 | 1 | 1 | NA | 8 (88.9) |
| Okoh et al., 1981 [93] | 1 | 1 | 0 | 1 | 1 | 1 | 1 | 1 | NA | 7 (77.8) |
| Okwuzu et al., 2014 [94] | 1 | 1 | 1 | 1 | 1 | 1 | 1 | 1 | NA | 8 (88.9) |
| Okwuzu et al., 2015 [95] | 1 | 0 | 1 | 1 | 1 | 1 | 1 | 1 | NA | 7 (77.8) |
| Olusi et al., 1996 [96] | 1 | 1 | 1 | 1 | 1 | 1 | 1 | 1 | NA | 8 (88.9) |
| Onadeko et al., 1992 [97] | 1 | 1 | 1 | 1 | 1 | 1 | 1 | 1 | NA | 8 (88.9) |
| Onakoya et al., 2012 [98] | 1 | 0 | 0 | 1 | 1 | 1 | 1 | 1 | NA | 6 (66.8) |
| Onosalkponome et al., 2019 [99] | 1 | 0 | 1 | 1 | 1 | 1 | 1 | 1 | NA | 7 (77.8) |
| Oshinaike et al., 2010 [100] | 1 | 1 | 0 | 1 | 1 | 1 | 1 | 1 | NA | 7 (77.8) |
| Osiyemi et al., 1985 [101] | 1 | 1 | 1 | 1 | 0 | 1 | 1 | 0 | NA | 6 (66.7) |
| Osunkalu et al., 2011 [102] | 1 | 1 | 0 | 1 | 1 | 1 | 1 | 1 | NA | 7 (77.8) |
| Oyinloye et al., 2014 [103] | 1 | 0 | 0 | 1 | 1 | 1 | 1 | 1 | NA | 6 (66.7) |
| Sanyaolu et al., 2016 [104] | 1 | 0 | 0 | 1 | 1 | 1 | 1 | 1 | NA | 6 (66.7) |
| Sixl et al., 1987 [105] | 0 | 1 | 0 | 1 | 1 | 1 | 1 | 1 | NA | 6 (66.7) |
| Sowemimo et al., 2018 [106] | 1 | 1 | 1 | 1 | 1 | 1 | 1 | 1 | NA | 7 (77.8) |
| Thomas et al., 1981 [107] | 1 | 0 | 0 | 1 | 1 | 1 | 1 | 1 | NA | 6 (66.7) |
| Uneke et al., 2005 [108] | 1 | 1 | 0 | 1 | 1 | 1 | 1 | 1 | NA | 7 (77.8) |
| Uneke et al., 2007 [109] | 0 | 1 | 0 | 1 | 1 | 1 | 1 | 1 | NA | 6 (66.7) |
| Uttah et al., 2013 [110] | 1 | 1 | 1 | 1 | 1 | 1 | 1 | 1 | NA | 8 (88.9) |
| Wiseman and Woodruff, 1970 [111] | 1 | 1 | 1 | 1 | 0 | 1 | 1 | 0 | NA | 6 (66.7) |
| Wokem et al., 2018 [112] | 1 | 1 | 0 | 1 | 1 | 1 | 1 | 1 | NA | 7 (77.8) |
| Yusuf et al., 2016 [113] | 1 | 1 | 1 | 1 | 1 | 1 | 1 | 1 | NA | 8 (88.9) |
| Yusuf and Airauhi, 2017 [114] | 1 | 1 | 1 | 1 | 1 | 1 | 1 | 1 | NA | 8 (88.9) |
