# Supplementary material for: Human Toxoplasma gondii infection in Nigeria: a systematic review and meta-analysis of data published between 1960 and 2019
Source: BMC Public Health. 2020 Jun 6;20:877. doi: 10.1186/s12889-020-09015-7 (PMC7276081; doi:10.1186/s12889-020-09015-7)

**EGGER’S REGRESSION ANALYSIS FOR STUDIES FROM NIGERIA**


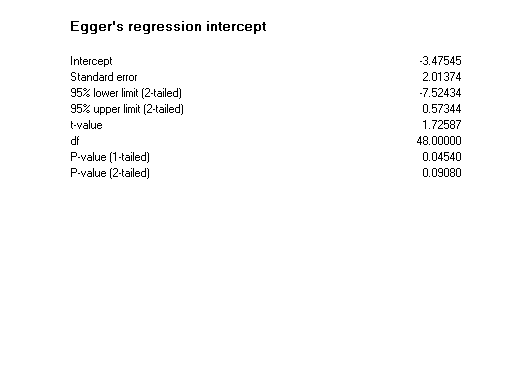


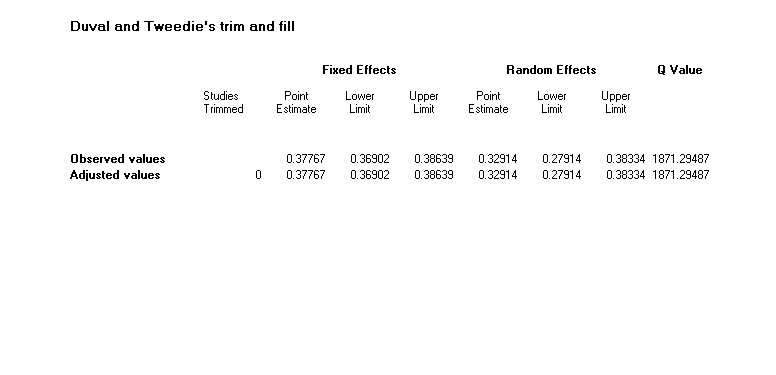


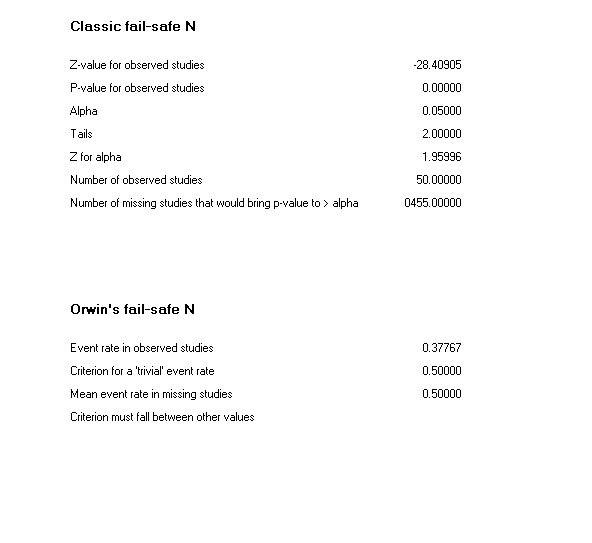


**EGGER’S REGRESSION ANALYSIS FOR STUDIES FROM NORTHERN NIGERIA**


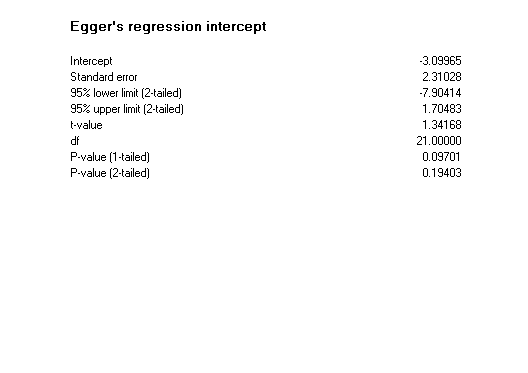


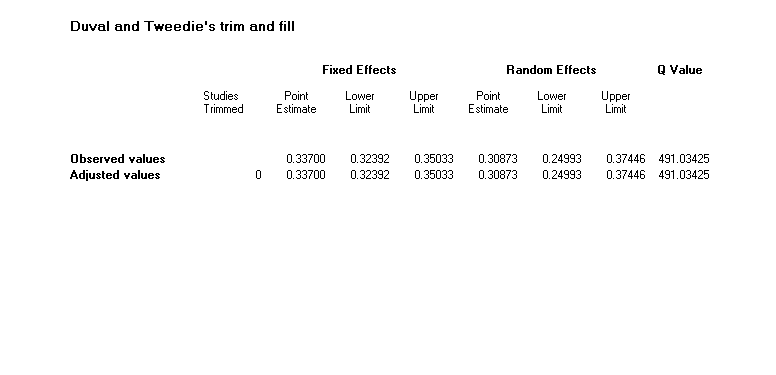


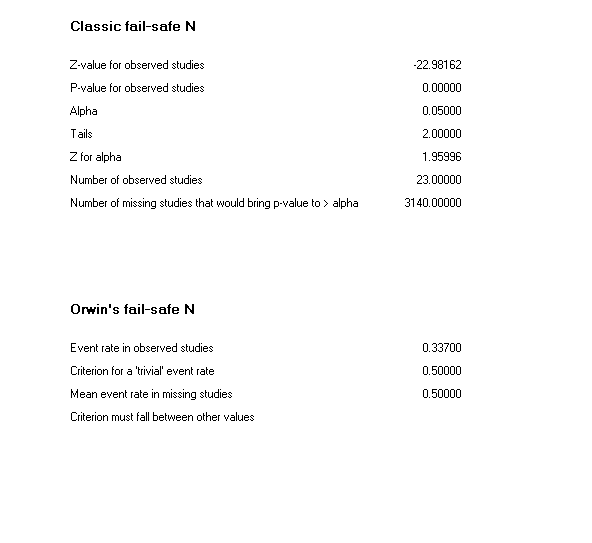


**EGGER’S REGRESSION ANALYSIS FOR STUDIES FROM SOUTHERN NIGERIA**


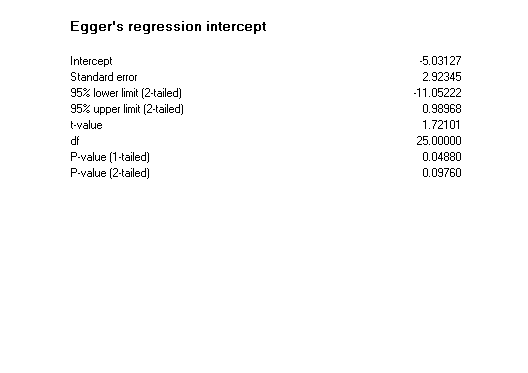


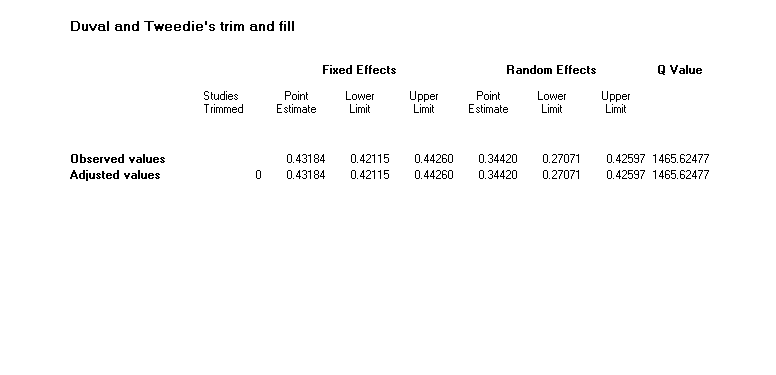


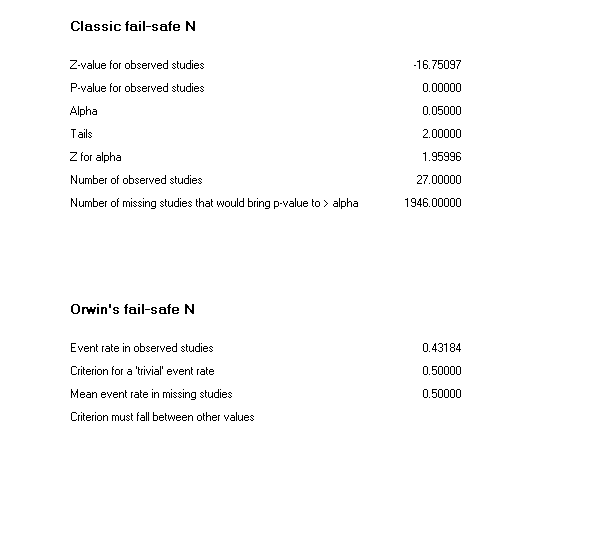

Supplement: Supplementary file 4 — Additional file 4. Results for Egger regression analysis. [file 12889_2020_9015_MOESM4_ESM.docx]
